# Diagnostic performance of the specific uptake size index for semi-quantitative analysis of I-123-FP-CIT SPECT: harmonized multi-center research setting versus typical clinical single-camera setting

**DOI:** 10.1186/s13550-019-0506-9

**Published:** 2019-05-07

**Authors:** Ralph Buchert, Catharina Lange, Timo S. Spehl, Ivayla Apostolova, Lars Frings, Cathrine Jonsson, Philipp T. Meyer, Sabine Hellwig

**Affiliations:** 1 Department for Diagnostic and Interventional Radiology and Nuclear Medicine, University Hospital Hamburg-Eppendorf, Martinistr. 52, 20246 Hamburg, Germany; 2 Department of Nuclear Medicine, Charité - Universitätsmedizin Berlin, corporate member of Freie Universität Berlin, Humboldt-Universität zu Berlin, and Berlin Institute of Health, Augustenburger Platz 1, 13353 Berlin, Germany; 3 Department of Nuclear Medicine, Medical Center - University of Freiburg, Faculty of Medicine, University of Freiburg, Freiburg, Germany; 4 Medical Radiation Physics and Nuclear Medicine, Imaging and Physiology, Karolinska University Hospital, Stockholm, Sweden; 5 Department of Psychiatry and Psychotherapy, Medical Center - University of Freiburg, Faculty of Medicine, University of Freiburg, Freiburg, Germany

**Keywords:** Dopamine transporter, SPECT, FP-CIT, Semi-quantitative analysis, Specific uptake size index, Specific binding ratio

## Abstract

**Introduction:**

The specific uptake size index (SUSI) of striatal FP-CIT uptake is independent of spatial resolution in the SPECT image, in contrast to the specific binding ratio (SBR). This suggests that the SUSI is particularly appropriate for multi-site/multi-camera settings in which camera-specific effects increase inter-subject variability of spatial resolution. However, the SUSI is sensitive to inter-subject variability of striatum size. Furthermore, it might be more sensitive to errors of the estimate of non-displaceable FP-CIT binding. This study compared SUSI and SBR in the multi-site/multi-camera (MULTI) setting of a prospective multi-center study and in a mono-site/mono-camera (MONO) setting representative of clinical routine.

**Methods:**

The MULTI setting included patients with Parkinson’s disease (PD, *n* = 438) and healthy controls (*n* = 207) from the Parkinson Progression Marker Initiative. The MONO setting included 122 patients from routine clinical patient care in whom FP-CIT SPECT had been performed with the same double-head SPECT system according to the same acquisition and reconstruction protocol. Patients were categorized as “neurodegenerative” (*n* = 84) or “non-neurodegenerative” (*n* = 38) based on follow-up data. FP-CIT SPECTs were stereotactically normalized to MNI space. SUSI and SBR were computed for caudate, putamen, and whole striatum using unilateral ROIs predefined in MNI space. SUSI analysis was repeated in native patient space in the MONO setting. The area (AUC) under the ROC curve for identification of PD/“neurodegenerative” cases was used as performance measure.

**Results:**

In both settings, the highest AUC was achieved by the putamen (minimum over both hemispheres), independent of the semi-quantitative method (SUSI or SBR). The putaminal SUSI provided slightly better performance with ROI analysis in MNI space compared to patient space (AUC = 0.969 vs. 0.961, *p* = 0.129). The SUSI (computed in MNI space) performed slightly better than the SBR in the MULTI setting (AUC = 0.993 vs. 0.991, *p* = 0.207) and slightly worse in the MONO setting (AUC = 0.969 vs. AUC = 0.976, *p* = 0.259). There was a trend toward larger AUC difference between SUSI and SBR in the MULTI setting compared to the MONO setting (*p* = 0.073). Variability of voxel intensity in the reference region was larger in misclassified cases compared to correctly classified cases for both SUSI and SBR (MULTI setting: *p* = 0.007 and *p* = 0.012, respectively).

**Conclusions:**

The SUSI is particularly useful in MULTI settings. SPECT images should be stereotactically normalized prior to SUSI analysis. The putaminal SUSI provides better diagnostic performance than the SUSI of the whole striatum. Errors of the estimate of non-displaceable count density in the reference region can cause misclassification by both SUSI and SBR, particularly in borderline cases. These cases might be identified by visual checking FP-CIT uptake in the reference region for particularly high variability.

## Introduction

Single-photon emission computed tomography (SPECT) with the I-123 labeled dopamine transporter (DAT) ligand FP-CIT is widely used for detection (or exclusion) of nigrostriatal degeneration in patients with clinically uncertain parkinsonian syndrome (PS) [1]. Semi-quantitative analysis of striatal FP-CIT uptake has the potential to support visual interpretation of the SPECT images [2, 3].

Semi-quantitative analysis in radionuclide imaging often aims at estimating the binding potential (BP), first defined by Mintun and co-workers as BP = Bmax / KD, where Bmax is the density of available binding sites (here DAT) and 1/KD is the affinity of the tracer (here FP-CIT) for the binding site [4]. The binding potential is a measure of the capacity of the region of interest (ROI) for specific binding of the tracer, that is, binding to the binding site of interest. More generally, a binding potential can be defined as the equilibrium concentration of specific binding to some other reference concentration [5]. The most widely used binding potential is the non-displaceable binding potential BPnd based on the concentration of the non-displaceable (by blocking of the binding site) tracer in the ROI as reference, i.e.,

1 $$ \mathrm{BPnd}=\mathrm{Cs}/\mathrm{Cnd}, $$

where Cs and Cnd are the equilibrium concentration of specifically bound and non-displaceable tracer in the ROI, respectively. Determination of BPnd requires dynamic imaging and arterial blood sampling for full tracer kinetic modeling. This is not feasible in clinical routine so that a number of methods have been developed to estimate BPnd from a single static scan during equilibrium (for FP-CIT approximately given between 3 and 6 h after i.v. injection [6]). The most widely used among these methods is the specific binding ratio (SBR) defined by the formula [7]

2 $$ \mathrm{SBR}=\left(\mathrm{C}-\mathrm{CR}\right)/\mathrm{CR}, $$

where C is the total count concentration in the striatal ROI, and CR is the total count concentration in a reference region (almost) void of DAT. The SBR according to formula (2) is based on the assumption that CR approximates the count concentration originating from non-displaceable FP-CIT binding in the striatal ROI (the difference C – CR then approximates the specific count density originating from FP-CIT bound to DAT in the striatal ROI). The striatal ROI for computation of the SBR usually anatomically delineates the striatum or the striatal subregion of interest such as caudate nucleus or putamen [8, 9]. A major limitation of the SBR according to formula (2) is the strong underestimation of the striatal FP-CIT concentration C in the SPECT images due to partial volume effects caused by limited spatial resolution of SPECT. The recovery of actual striatal FP-CIT concentration in SPECT images typically is only about 50% if no correction for partial volume and other degrading effects is performed [10–12]. In addition to strong underestimation of the SBR, partial volume effects cause additional variability. This is due to the fact that the magnitude of the partial volume effect strongly depends on the size and shape of the striatum and the spatial resolution in the reconstructed SPECT image. Spatial resolution varies between SPECT cameras depending on both, hardware and acquisition/reconstruction protocol. The additional variability of the SBR associated with the use of different SPECT cameras negatively impacts the utility of the SBR, particularly in multi-site and in single-site/multi-camera settings or when normal values and cut-offs obtained from healthy subjects at one site are to be used at other sites [13, 14]. Only in an ideal situation in which all main causes of inaccuracy and imprecision of SPECT (attenuation, scatter, partial volume effects, statistical noise) were properly dealt with, the true SBR value would be obtained with any SPECT camera and reduction of inter-camera variability would naturally follow from accuracy.

The specific uptake size index (SUSI) has been proposed to improve accuracy of semi-quantitative analysis in FP-CIT SPECT in practice [7, 15, 16]. It has been thoroughly validated in phantom studies where true count densities are known [15, 17]. The SUSI eliminates partial volume effects by replacing *count density* in the striatal ROI by a measure of *total counts* in the ROI. More precisely, the SUSI is defined as

3 $$ \mathrm{SUSI}=\left(\mathrm{T}-\mathrm{CR}\times \mathrm{V}\right)/\mathrm{CR}, $$

where T is the total number of counts originating from a large striatal ROI, V is the volume of the striatal ROI, and CR is the count concentration in the reference region.

The striatal ROI for the computation of the SUSI is chosen sufficiently large around the striatum to guarantee that all counts originating from the striatum are detected within this ROI. Assuming that image reconstruction is “activity conserving” (that is, counts are neither lost nor artificially produced by the reconstruction, although limited spatial resolution can cause counts originating from the striatum to be localized outside of the striatum), summing the activity over all voxels within the large striatum ROI collects all counts originating from the striatum. This holds true independently of the spatial resolution in the reconstructed SPECT image as long as the ROI is large enough to encompass all counts originating from the striatum. Thus, the SUSI is independent of spatial resolution [15]. The SUSI approach can be seen as a method for partial volume correction of the SBR. This is due to the fact that SBR = SUSI / Vs, where Vs is the actual volume of the striatum, as is easily derived from Eqs. (2) and (3) [15].

The SUSI has been used successfully for the quantitative characterization of age effects on DAT availability in healthy subjects from the European normal control database of FP-CIT SPECT (ENC-DAT) [18]. A total of 13 sites using ten different SPECT camera models contributed to the ENC-DAT study.

The SUSI is typically computed in the original SPECT images in patient space after manual reorientation [7, 15, 16]. A limitation of the SUSI computed in patient space is that it also depends on the size of the striatum, not via partial volume effects as the SBR, but via the total amount of specifically bound FP-CIT that increases with increasing size of the striatum (assuming constant DAT concentration). Thus, inter-subject variability of striatum size causes additional variability of the SUSI that might limit its power to detect nigrostriatal degeneration. The impact of inter-subject variability of striatum size on the SUSI might be eliminated by concentration-preserving stereotactical normalization of the SPECT images into an anatomical standard space prior to ROI analysis. Inter-subject variability of partial volume effects cannot be eliminated this way.

A recent study suggested the SUSI to be more sensitive than the SBR to errors of the estimate of non-displaceable count density in the striatum by the count density CR in the reference region [19]. In both methods, SBR and SUSI, the reference region is used to correct the striatal signal for non-displaceable “background” in the striatum ROI that dilutes the effect of nigrostriatal degeneration in these measures if not corrected for. It is evident that the background contribution increases when the ROI size increases beyond the anatomical boundaries of the striatum. Thus, the relative magnitude of the correction for non-displaceable background increases with increasing ROI size. As a consequence, the computed measure of specific striatal binding (SBR or SUSI) might be more sensitive to errors (including statistical noise) of the estimate of non-displaceable FP-CIT binding the larger the striatal ROI.

Aim of the present study was to compare the SUSI and the SBR with respect to detection of nigrostriatal degeneration in (i) a sample of patients with PS in whom FP-CIT SPECT has been performed with the same SPECT camera using the same acquisition protocol with radius of rotation within very tight limits [20] and the same reconstruction protocol (mono-site/mono-camera (MONO) setting) and (ii) in the multi-site/multi-camera (MULTI) setting of the Parkinson Progression Markers Initiative (PPMI) [21]. Inter-subject variability of spatial resolution in the SPECT images most likely was larger in the MULTI setting compared to the MONO setting (due to camera-specific variability of spatial resolution in the MULTI setting). The primary hypothesis put to test was that the diagnostic performance of the SUSI relative to the SBR is better in the MULTI setting compared to the MONO setting (due to its stability with respect to varying spatial resolution in the SPECT images), particularly when the SUSI is computed after stereotactical normalization (in order to eliminate inter-subject variability of striatum size) rather than in raw images in patient space [7]. The secondary hypothesis put to test was that the variability of the count density in the reference region impacts the diagnostic performance of both SUSI and SBR, and that the effect is larger for the SUSI than for the SBR.

## Materials and methods

### Mono-site/mono-camera setting

One hundred twenty-two patients in whom FP-CIT SPECT had been performed for detection (or exclusion) of nigrostriatal degeneration as part of routine clinical work-up were recruited retrospectively from the patient database of the Department of Nuclear Medicine of the University Medical Center Freiburg. The patients were categorized into two groups: “neurodegenerative PS” and “non-neurodegenerative PS.” The neurodegenerative group (*n* = 84, 39 females, 67.3 ± 10.0 years) comprised (i) the Lewy body disease spectrum (*n* = 60) including Parkinson’s disease (PD, *n* = 41), PD dementia (PDD, *n* = 5), and dementia with Lewy bodies (DLB, *n* = 14), and (ii) atypical parkinsonian syndromes (APS, *n* = 24) including multiple systems atrophy (MSA-P and MSA-P/C, *n* = 7), progressive supranuclear palsy (PSP, *n* = 13), and corticobasal degeneration (CBD, *n* = 4). The non-neurodegenerative group (*n* = 38, 15 females, 70.0 ± 8.0 years) comprised (i) essential tremor (*n* = 2), (ii) vascular parkinsonism (VaP, *n* = 7), (iii) drug-induced parkinsonism (*n* = 11), (iv) psychogenic parkinsonism (*n* = 1), (v) possible Alzheimer’s disease (AD, *n* = 11), and (vi) normal pressure hydrocephalus (NPH, *n* = 6). The clinical diagnoses were established by a movement disorder specialist based on current consensus criteria and review of all relevant medical charts and clinical follow-up data (mean follow-up 26.8 ± 14.5 months). Patients with other than the listed diagnosis were excluded.

FP-CIT SPECT had been performed according to common guidelines [22, 23]. The same double-head SPECT system (Siemens E.CAM) and the same acquisition protocol had been used in all patients (thyroid uptake blocked with sodium perchlorate, 3 h uptake period after intravenous bolus injection of 193 ± 8 MBq FP-CIT; low-energy high-resolution parallel-hole collimators, 60 projections of 30 s duration with each head along a scan arc of 180° (i.e. 3° angular sampling), radius of rotation = 13.5 ± 0.3 cm, energy window 144–168 keV, acquisition matrix 128 × 128, zoom factor 1.23). In order to ensure consistent image reconstruction in all patients, projection data were exported from the archive and retrospectively reconstructed by three-dimensional ordered subset expectation maximization (OSEM) with resolution recovery using the Flash3D algorithm of the scanner software. The following parameter settings were used as previously proposed for FP-CIT SPECT specifically with the E.CAM camera to provide a good compromise between delineation of striatal substructures for visual inspection, recovery of striatal tracer uptake, and noise of non-displaceable tracer uptake in the reference region for semi-quantitative analysis [24]: 8 iterations, 8 subsets, postfiltering with a Gaussian kernel with 8 mm full-width-at-half-maximum (FWHM). Uniform post reconstruction attenuation correction was performed according to Chang (first order, broad-beam attenuation coefficient μ = 0.12/cm); no scatter correction was performed. Voxel size was 3.9 × 3.9 × 3.9 mm^3^.

### Multi-site/multi-camera setting

Data were obtained from the Parkinson’s Progression Markers Initiative (PPMI) database (https://www.ppmi-info.org/access-data-specimens/download-data/) [21]. Up-to-date information on the PPMI is available at www.ppmi-info.org. The PPMI is a longitudinal, multi-center study that aims to assess the progression of clinical features, imaging, and biologic markers in patients with (idiopathic) PD and healthy control (HC) subjects. All PD patients were in an early stage of the disease (diagnosis of PD within the last 2 years prior to screening). Details of the eligibility criteria are given at http://www.ppmi-info.org/wp-content/uploads/2014/01/PPMI-AM7-Protocol.pdf.

All FP-CIT scans available from the PPMI database on 22nd November 2017 were downloaded in DICOM format, independent of subgroup and visit (*n* = 1710). The analyses of the present study included the first FP-CIT SPECT of all HC subjects and all PD patients, that is, the analyses included also FP-CIT SPECT at an “unscheduled” visit if FP-CIT SPECT at the screening visit was not available. Furthermore, the analyses included not only “regular” HC subjects and “regular” PD patients but also subjects who (i) declined participation in the PPMI study after the screening visit but before inclusion in the study (*n* = 20), or (ii) withdrew agreement after inclusion in the study (*n* = 78), or (iii) were excluded from participation in the study due to a reason not related to FP-CIT SPECT (*n* = 23). This resulted in 656 FP-CIT scans. Visual inspection resulted in exclusion of 11 of these scans: three HC scans were excluded because of clearly reduced striatal FP-CIT uptake (PPMI-ID 3221, 3478, 4095); eight PD scans were excluded because of clearly normal FP-CIT uptake in the striatum (3027, 3289, 3290, 3534, 3618, 3623, 3660, 3863). This resulted in the inclusion of a total of 645 FP-CIT SPECTs: 438 of PD patients and 207 of HC subjects.

FP-CIT SPECT data had been acquired at 24 different centers using different SPECT camera models. All centers had been qualified for participation in the study by an image center qualification process including a technical set up visit to optimize the acquisition and reconstruction protocol for the specific SPECT system to be used in the study [21]. The target dose of FP-CIT was 185 MBq (allowed range 110–185 MBq) and SPECT acquisition was to be started 4 ± 0.5 h after i.v. administration of FP-CIT (PPMI imaging protocol at http://www.ppmi-info.org/study-design/research-documents-and-sops/). Raw projection data had been transferred to the PPMI imaging core lab for central image reconstruction using an OSEM algorithm on a HERMES workstation (Hermes Medical Solutions, Stockholm, Sweden) [25]. PMOD (PMOD Technologies, Zurich, Switzerland) had been used for attenuation correction. Ellipses were drawn on the images and 0th order Chang attenuation correction was applied using a site-specific μ empirically derived from phantom data. Standard 3D Gaussian post-smoothing (6.0 mm FWHM) was applied. Only preprocessed images that have been stereotactically normalized into the anatomical space of the Montreal Neurological Institute (MNI) were available for download. The images were in DICOM format with 91 × 109 × 91 cubic voxels of 2 mm edge length.

### Semi-quantitative analyses

All semi-quantitative analyses were performed fully automatically using a MATLAB script.

**Fig. 1:**
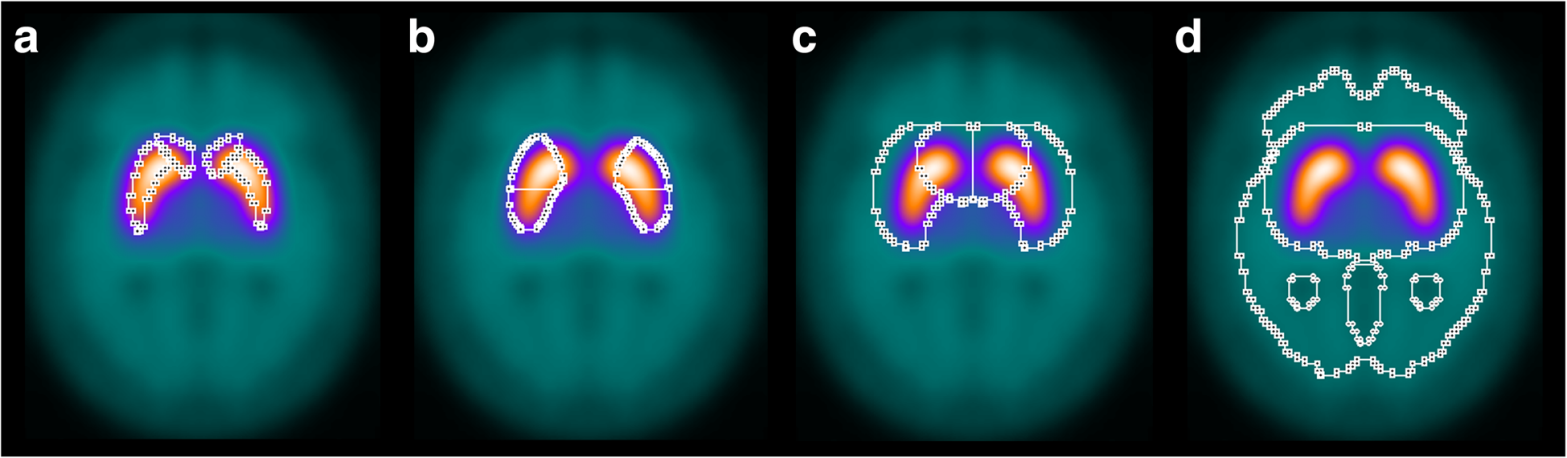
Anatomical caudate and putamen ROIs from the AAL atlas (**a**), custom-made anatomical ROIs of anterior and posterior putamen (**b**), large ROIs of caudate and putamen (**c**), and reference region (**d**). All ROIs are three-dimensional. The ROIs are overlaid to the custom-made FP-CIT template used as target for stereotactical normalization. The anatomical ROIs in (**a**) and (**b**) were used for computation of the SBR. The large ROIs in (**c**) were used for computation of the SUSI. The custom-made FP-CIT template was obtained by averaging the stereotactically normalized images of 94 visually normal FP-CIT SPECT scans

SBR was computed according to formula (2) with ROI analysis in MNI space. For this purpose, individual FP-CIT SPECTs were transformed (affine, that is, without warping) into MNI space using the Statistical Parametric Mapping software package (version SPM12) and a custom-made FP-CIT template (Fig. 1). Unilateral ROIs for caudate, putamen, and entire striatum (= union of caudate and putamen ROI) predefined in MNI space by the automatic anatomic labeling (AAL) atlas [26] available in the PickAtlas of the Wake Forest University were used (Fig. 1a) [27]. In addition, custom-made unilateral ROIs for anterior and posterior putamen manually predefined in MNI space were tested (Fig. 1b). Count concentration within a ROI was characterized by the average intensity over all voxels within the ROIs. The 75th percentile of the count density in a reference region comprising the whole brain without striata, thalamus, and brain stem was used as estimate of non-displaceable count density (Fig. 1d) [28].

The SUSI was computed according to formula (3) with ROI analysis in MNI space. It was computed separately for unilateral caudate and unilateral putamen using large three-dimensional unilateral ROIs predefined in MNI space (Fig. 1c). The union of large caudate and large putamen ROI was used to compute the SUSI of the entire unilateral striatum. In the MONO setting, SUSI analysis was repeated in native patient space. For this purpose, the FP-CIT template was stereotactically normalized into patient space (affine transformation). The resulting transformation was used to map the ROIs from MNI space to the patient’s FP-CIT SPECT.

### Statistical analyses

SUSI and SBR were tested for identification of “neurodegenerative PS” in the MONO setting and for identification of PD patients in the MULTI setting using the minimum over both hemispheres for both semi-quantitative measures. The area (AUC) under the receiver operating characteristic (ROC) curve was employed as performance measure. Total accuracy, sensitivity, and specificity were computed with the cut-off fixed by Youden’s criterion [29].

In order to test for potential impact of individual putamen size on the diagnostic performance of the putaminal SUSI computed in patient space, the volume of the AAL putamen ROI after transformation into patient space was used as measure of individual putamen size (mean of both hemispheres). Putamen size was compared between true positive, true negative, false positive, and false negative cases as classified by the SUSI computed in patient space. Univariate analysis of variance was used for this purpose.

In order to test the secondary hypothesis, that is, the impact of the uncertainty of the estimate of non-displaceable count density by the 75th percentile of voxel intensities in the reference region on the diagnostic performance of SUSI and SBR, the interquartile range (IQR) of voxel intensities in the reference region relative to the 75th percentile of voxel intensities in the reference region was used as uncertainty measure (relative IQR). First, relative IQR was compared between correctly classified scans (true positive or true negative) and incorrectly classified scans (false positive or false negative) using unpaired *t* tests. This was done separately for SUSI and SBR. Second, SUSI and SBR were compared with respect to the difference of the relative IQR between correctly classified scans and incorrectly classified scans using univariate analysis of variance of relative IQR with both, correctness of the SUSI-based classification and correctness of the SBR-based classification as fixed factors. Cut-offs fixed by Youden’s criterion were used to categorize scans as correctly or incorrectly classified.

In quantitative terms, the primary hypothesis of the study (the diagnostic performance of the SUSI relative to the SBR is better in the MULTI setting than in the MONO setting) states that (AUC_SUSI_ – AUC_SBR_) (MULTI) > (AUC_SUSI_ – AUC_SBR_) (MONO), where AUC_SUSI_ is the AUC of the putaminal SUSI and AUC_SBR_ is the AUC of the putaminal SBR. Bootstrapping with 100,000-fold resampling was used to estimate the distribution of (AUC_SUSI_ – AUC_SBR_), separately for the MULTI and the MONO setting. Then, the distribution of (AUC_SUSI_ – AUC_SBR_) was compared between the MULTI and the MONO setting by generating 10,000 random pairs of (AUC_SUSI_ – AUC_SBR_) (MULTI) and (AUC_SUSI_ – AUC_SBR_) (MONO) from their respective distributions and counting the number of random pairs that fulfilled the alternative hypothesis (AUC_SUSI_ – AUC_SBR_) (MULTI) ≤ (AUC_SUSI_ – AUC_SBR_) (MONO).

## Results

Fully automatic affine transformation of FP-CIT SPECT from patient space into the anatomical MNI template space or vice versa worked properly in all cases according to visual inspection. Reliable spatial transformation is required for automatic semi-quantitative analysis with standard ROIs predefined in template space.

### Mono-site/mono-camera setting

**Fig. 2:**
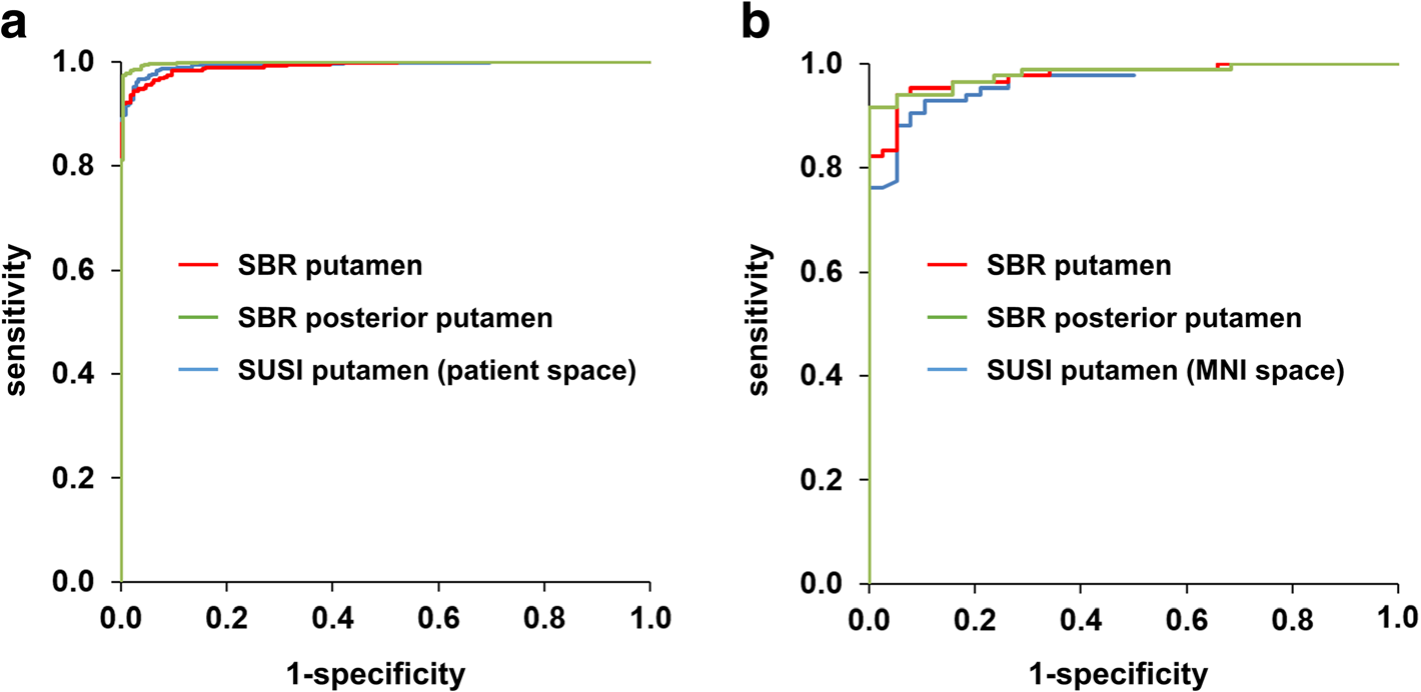
ROC curves for identification of neurodegenerative etiology of parkinsonism (mono-site/mono-camera setting, **a**) or PD (multi-site/multi-camera setting, **b**)

SUSI and SBR showed better diagnostic performance with the putamen as region of interest compared to caudate and whole striatum (Table 1). The AUC provided by the putaminal measures was largest for the SBR of the posterior putamen (AUC = 0.981) followed by SBR of the whole putamen (AUC = 0.976). The putaminal SUSI showed slightly worse performance, particularly when computed in patient space (AUC = 0.961, DeLong test *p* = 0.036 compared to SBR of the whole putamen; Table 1, Fig. 2a). Stereotactical normalization prior to ROI analysis improved the performance of the putaminal SUSI from AUC = 0.961 to AUC = 0.969, but the difference did not reach statistical significance (*p* = 0.129, Table 1). Classification of patients (as “neurodegenerative PS” or “non-neurodegenerative PS”) was incorrect in 7 (5.7%), 12 (9.8%), and 8 (6.6%) of the 122 patients when based on putaminal SBR, putaminal SUSI computed in patient space, or putaminal SUSI computed in MNI space, respectively (Table 1).

**Table 1:** Area (AUC) under the ROC curve, cut-off (based on Youden’s criterion), and resulting total accuracy, sensitivity, and specificity for identification of neurodegenerative etiology of parkinsonism (mono-site/mono-camera setting) or PD (multi-site/multi-camera setting). (Cau = caudate, Put = putamen, Str = striatum, ant = anterior, post = posterior)

		SBR					SUSI in MNI space			SUSI in patient space		
		Cau	Put	ant Put	post Put	Str	Cau	Put	Str	Cau	Put	Str
Mono-camera	AUC (95% CI)	0.636^bbb,ccc^ (0.526–0.746)	0.976^c^ (0.954–0.999)	0.935^b^ (0.891–0.980)	0.981^c^ (0.960–1.000)	0.925 (0.877–0.973)	0.764^aaa^ (0.673–0.855)	0.969 (0.943–0.996)	0.927 (0.881–0.974)	0.765^aaa^ (0.675–0.855)	0.961^a^ (0.930–0.993)	0.924 (0.874–0.974)
	Cut-off	1.829	2.383	1.866	1.776	2.051	16.418	16.974	34.902	12.913	8.371	23.653
	Accuracy	0.672	0.943	0.885	0.943	0.877	0.705	0.934	0.877	0.770	0.902	0.877
	Sensitivity	0.762	0.940	0.857	0.917	0.881	0.643	0.929	0.869	0.821	0.881	0.881
	Specificity	0.474	0.947	0.947	1.000	0.868	0.842	0.947	0.895	0.658	0.947	0.868
Multi-camera	AUC (95% CI)	0.884^bbb^ (0.856–0.911)	0.991 (0.987–0.996)	0.966^bbb^ (0.954–0.978)	0.998^b^ (0.996–1.000)	0.979 (0.971–0.988)	0.920^aaa^ (0.898–0.942)	0.993 (0.989–0.998)	0.978 (0.969–0.987)	–	–	–
	Cut-off	1.781	2.068	1.776	1.597	1.960	21.229	14.799	39.171	–	–	–
	Accuracy	0.780	0.953	0.898	0.980	0.930	0.839	0.964	0.933	–	–	–
	Sensitivity	0.719	0.943	0.872	0.973	0.916	0.822	0.961	0.945	–	–	–
	Specificity	0.908	0.976	0.952	0.995	0.961	0.874	0.971	0.908	–	–	–

Statistical testing was restricted to caudate versus caudate, putamen versus putamen, and striatum versus striatum

^a/aa/aaa^DeLong test *p* < 0.05/0.01/0.005 compared to SBR

^b/bb/bbb^DeLong test *p* < 0.05/0.01/0.005 compared to SUSI in MNI space

^c/cc/ccc^DeLong test *p* < 0.05/0.01/0.005 compared to SUSI in patient space

**Fig. 3:**
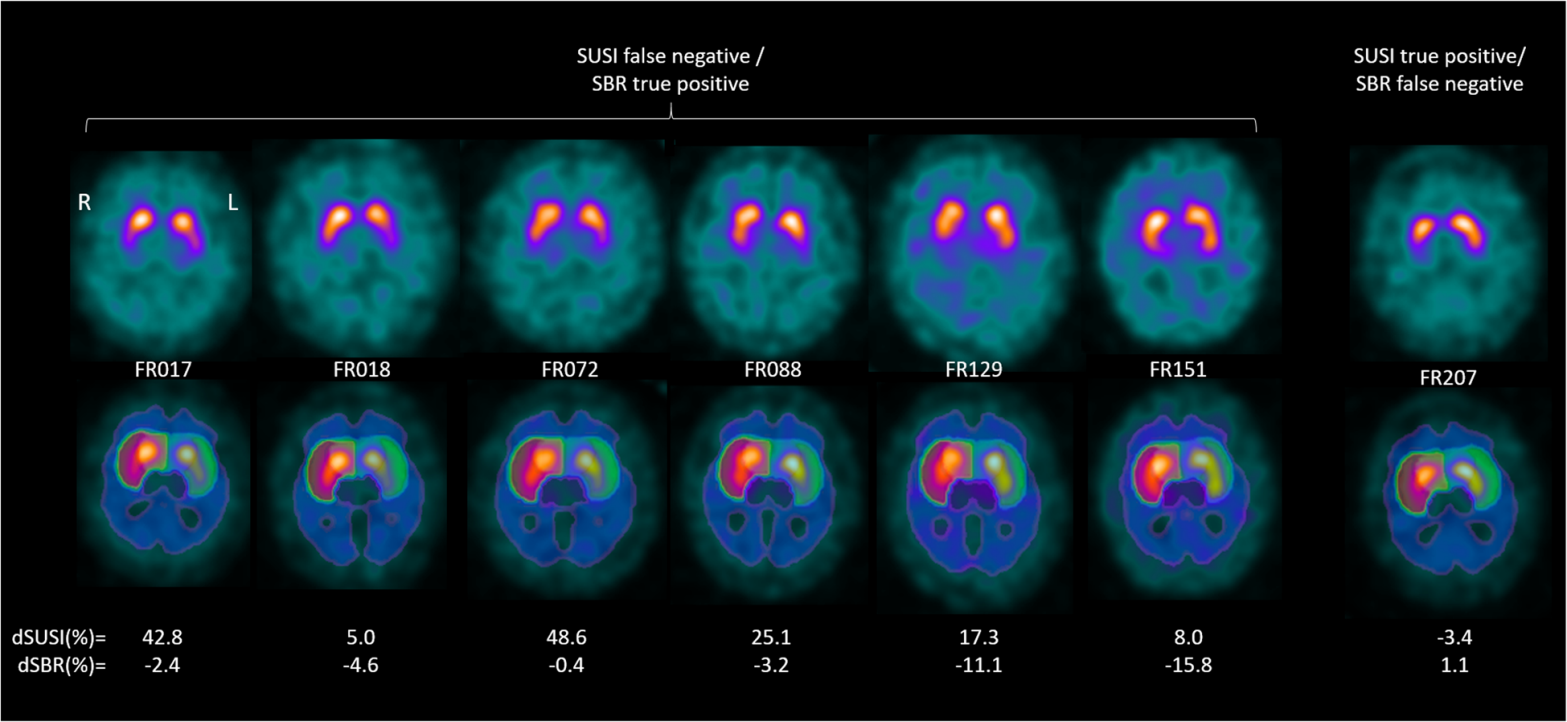
FP-CIT SPECT images from the mono-site/mono-camera setting with discrepant classification as “neurodegenerative PS” or “non-neurodegenerative PS” based on the putaminal SUSI computed in patient space versus classification based on the putaminal SBR. The upper row shows transversal slices through the striatum in patient space. The lower row shows the same transversal slice with the transformed mask of caudate, putamen and the reference region overlaid. dSUSI = 100*(individual SUSI – SUSI cutoff) / SUSI cutoff, dSBR = 100*(individual SBR – SBR cutoff) / SBR cutoff

Classification based on putaminal SUSI computed in patient space or putaminal SBR was discrepant in seven patients (5.7%). All these discrepant cases had “neurodegenerative PS” and presented with borderline findings in FP-CIT SPECT (Fig. 3). Classification based on putaminal SUSI computed in patient space was correct (true positive) and SBR-based classification was incorrect (false negative) in one of these patients. Classification based on putaminal SUSI computed in patient space was incorrect (false negative) and SBR-based classification was correct (true positive) in the remaining six patients.

**Fig. 4:**
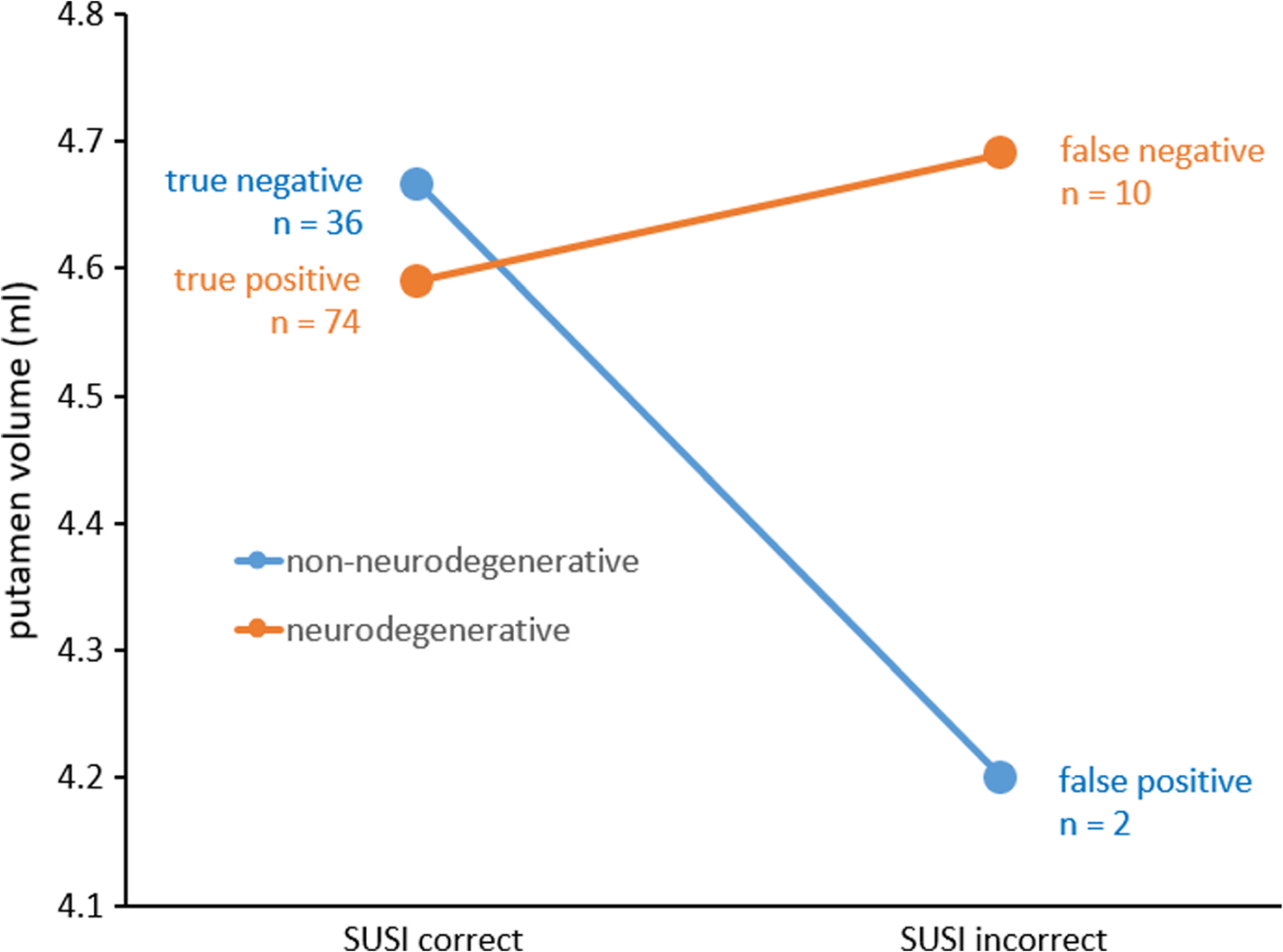
Comparison of individual putamen volume between true positive and false negative cases and between true negative and false positive cases as classified by the SUSI computed in patient space. None of the differences reached statistical significance

Putamen volume was smaller in the two false positive cases compared to the 36 true negative cases based on putaminal SUSI in patient space; it was larger in the ten false negative cases compared to the 74 true positive cases (Fig. 4). However, neither of the two differences reached statistical significance according to analysis of variance with standard of truth (“neurodegenerative PS” versus “non-neurodegenerative PS,” *p* = 0.314) and correctness of patient space SUSI (true positive or true negative versus false positive or false negative, *p* = 0.372) as fixed factors (standard of truth * correctness interaction *p* = 0.170).

The relative IQR of voxel intensities in the reference region was not different between the 12 patients with incorrect classification based on patient space SUSI compared to the remaining 110 patients (0.164 ± 0.012 versus 0.173 ± 0.024, *t* test *p* = 0.199).

### Harmonized multi-site/multi-camera setting

In the multi-site/multi-camera setting, the SUSI was computed in MNI space only, because the PPMI provides only preprocessed FP-CIT SPECT images in MNI space, the original FP-CIT images in patient space are not available. Thus, all SUSI results in the multi-site/multi-camera setting refer to the SUSI computed in MNI space.

In the harmonized MULTI setting, too, the putamen achieved the highest performance with both SUSI and SBR (Table 1). The AUC of the SUSI increased to AUC = 0.993 when the putamen was used as region of interest from AUC = 0.978 for the whole striatum (*p* < 0.001).

The AUC provided by the putaminal measure was largest for the SBR of the posterior putamen (AUC = 0.998), followed by the SUSI (AUC = 0.993) and the SBR of the whole putamen (AUC = 0.991) (Fig. 2b). The AUC difference between SUSI and SBR of the whole putamen did not reach statistical significance (*p* = 0.207). Classification of subjects (as PD or HC) was incorrect in 30 (4.7%) and 23 (3.6%) of the 645 subjects when based on putaminal SBR or putaminal SUSI, respectively (Table 1).

The relative IQR of voxel intensities in the reference region was higher in the 23 subjects with incorrect SUSI-based classification compared to the remaining 622 subjects with correct SUSI-based classification (0.15713 ± 0.03098 versus 0.14390 ± 0.02259, *t* test *p* = 0.007). The same was true for the 30 subjects with incorrect SBR-based classification compared to the remaining 615 subjects (0.15474 ± 0.02612 versus 0.14387 ± 0.02278, *t* test *p* = 0.012).

**Fig. 5:**
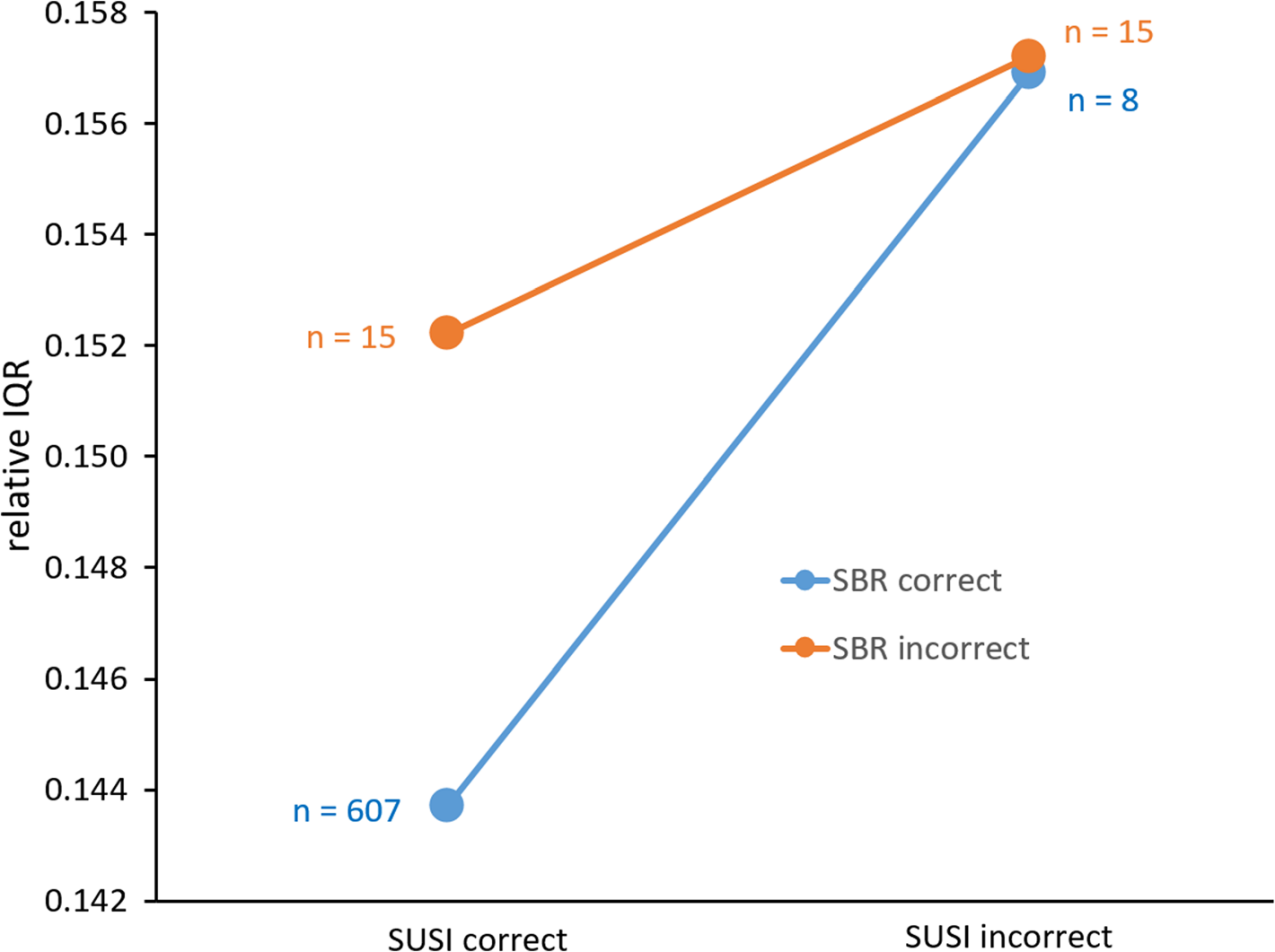
Interquartile range (IQR) relative to the 75th percentile of the voxel intensity in the reference region according to correctness of SUSI-based and SBR-based classification of the PPMI FP-CIT images as PD or HC (“correct” = true positive or true negative, “incorrect” = false positive or false negative). The relative IQR is as measure of the uncertainty of the estimate of the non-displaceable count density by the 75th percentile of the voxel intensity in the reference region

Classification based on putaminal SUSI or on putaminal SBR was discrepant in 23 of the 645 patients (3.6%). The relative IQR of voxel intensities in the reference region was higher in the 23 subjects with discrepant classification compared to the remaining 622 subjects with concordant classification (0.154 ± 0.028 versus 0.144 ± 0.023, *t* test *p* = 0.044). SUSI-based classification was correct and SBR-based classification was incorrect in two HC subjects and 13 PD patients. SUSI-based classification was incorrect and SBR-based classification was correct in three HC subjects and 5 PD patients. The difference of relative IQR between incorrectly classified scans and correctly classified scans based on the SUSI was larger for scans with correct SBR-based classification (0.157 ± 0.037, *n* = 8, versus 0.144 ± 0.023, *n* = 607) compared to scans with incorrect SBR-based classification (0.157 ± 0.029, *n* = 15, versus 0.152 ± 0.024, *n* = 15) (Fig. 5). However, the difference was not significant according to univariate analysis of variance of relative IQR with correctness of the SUSI-based classification and the correctness of the SBR-based classification as fixed factors (SUSI correctness * SBR correctness interaction *p* = 0.482).

### SUSI versus SBR in harmonized multi-site versus mono-camera setting

The putaminal SUSI performed slightly worse than the putaminal SBR in the MONO setting (AUC = 0.969 versus 0.976, *p* = 0.259) and slightly better in the MULTI setting (AUC = 0.993 versus 0.991, *p* = 0.207).

**Fig. 6:**
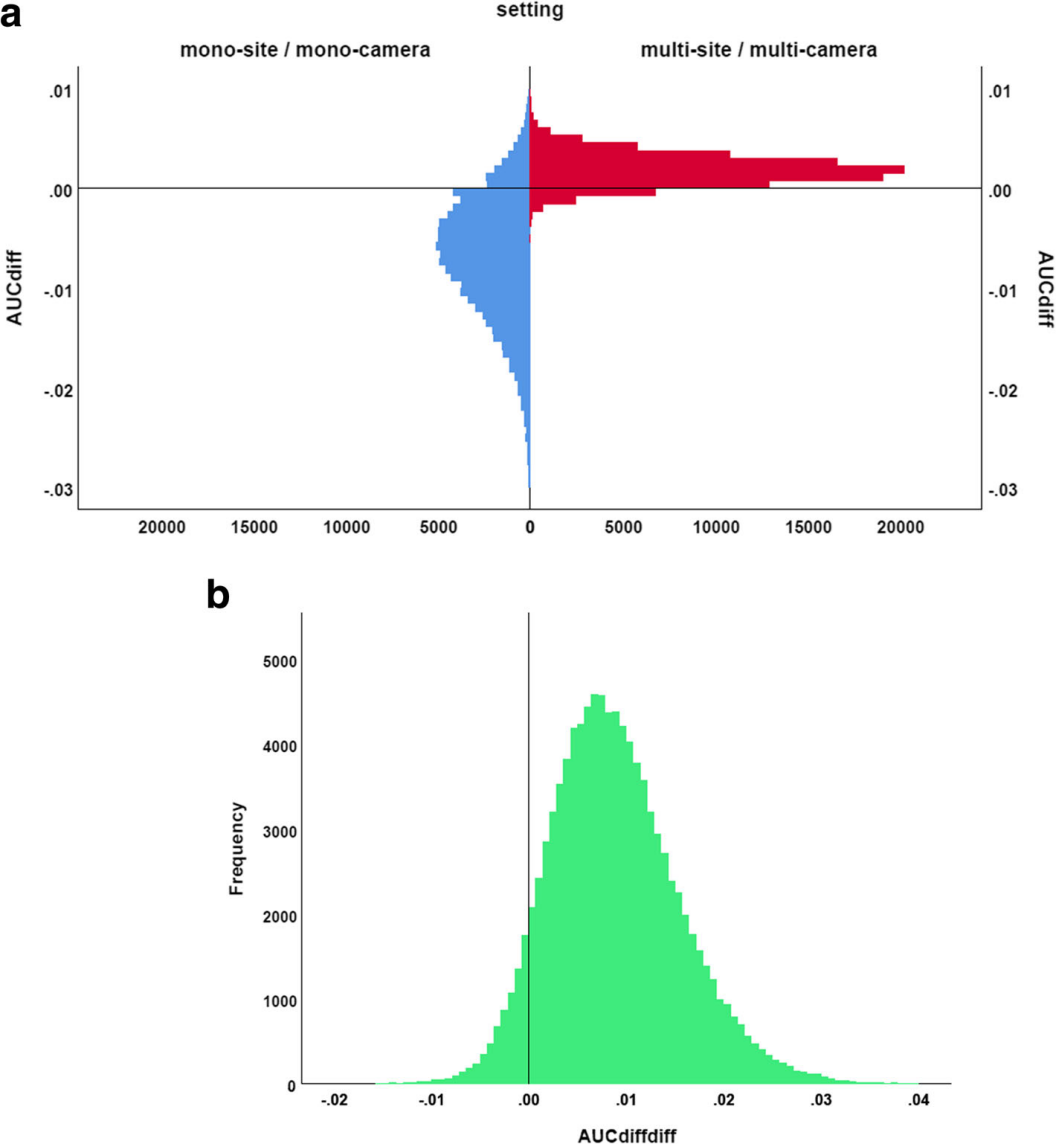
Distribution of the performance difference AUC_diff_ = (AUC_SUSI_ – AUC_SBR_) between putaminal SUSI and putaminal SBR in the mono-site/mono-camera (MONO) setting and in the multi-site/multi-camera (MULTI) setting (**a**), and distribution of the double difference AUC_diffdiff_ = AUC_diff_ (MULTI) – AUC_diff_ (MONO) (**b**). All distributions were estimated by bootstrapping with 100,000-fold resampling

The distribution of the difference (AUC_SUSI_ − AUC_SBR_) in the MULTI setting and in the MONO setting estimated by bootstrapping is shown in Fig. 6. Mean (AUC_SUSI_ − AUC_SBR_) was 0.002 (95% CI -0.001–0.005) in the MULTI setting and -0.007 (-0.021–0.004) in the MONO setting. The sign test revealed a trend toward larger (AUC_SUSI_ – AUC_SBR_) in the MULTI setting compared to the MONO setting (*p* = 0.073).

## Discussion

SUSI and SBR showed better classification performance with the putamen as region of interest compared to the whole striatum. The difference was highly significant for both SUSI and SBR (e.g., multi-site/multi-camera setting: putaminal SUSI = 0.993, striatal SUSI = 0.978, *p* < 0.001, Table 1). The fraction of misclassified cases was almost twice as large for the SUSI of the whole striatum compared to the putaminal SUSI (6.7% versus 3.6% in the MULTI setting, Table 1). Thus, the SUSI should be used with the putamen as striatal region of interest. This might appear in conflict with the rationale of the SUSI at first sight, because there is some loss of putaminal counts and some contamination by counts from the caudate at the boundary between putamen and caudate ROI. This probably explains that all previous studies used the SUSI with the whole striatum as region of interest [7, 15, 16, 18, 19]. Furthermore, the putaminal SUSI depends on the definition of the putamen ROI, in contrast to the striatal SUSI that is rather independent of the striatum ROI as long as it is large enough [15]. However, the SBR has the same limitations: the SBR of the (whole) putamen depends on the putamen ROI, and it is affected by spill-out and spill-in at the boundary between putamen and caudate ROI. These limitations, therefore, should not prevent use of the putaminal SUSI, considering the relevant improvement in diagnostic accuracy it provides compared to the conventional SUSI of the whole striatum. The large putamen ROI used for SUSI analysis in the present study excluded part of the anterior putamen in order to reduce contamination of the putaminal SUSI by counts from the caudate (Fig. 1c). As a result, the contribution of the posterior putamen was pronounced in the putaminal SUSI. This probably contributed to the excellent performance of the putaminal SUSI in the multi-site/multi-camera setting.

In the MONO setting, putaminal SUSI computed in patient space performed slightly worse than the SUSI computed in MNI space (AUC = 0.961 versus 0.969, *p* = 0.129, total accuracy = 90.2% versus 93.4%, Table 1). Putamen volume was smaller in the two false positive cases compared to the 36 true negative cases based on putaminal SUSI in patient space; it was larger in the ten false negative cases compared to the 74 true positive cases (Fig. 4). This is in line with the fact that the putaminal SUSI increases with increasing putamen size so that the SUSI tends to overestimate putaminal DAT concentration in patients with large putamen and to underestimate putaminal DAT density in patients with small putamen. This can be avoided by scaling the putaminal SUSI to individual putamen size estimated from individual high resolution MRI which, however, is not always available in clinical routine. Alternatively, inter-subject variability of putamen size can be reduced by concentration-preserving stereotactical normalization, that is, stereotactical normalization without modulation to account for the amount of local expansion/contraction that typically is used in MRI-based morphometry to guarantee that regional brain volume is the same in anatomical standard space as in the original image in patient space. Based on the findings of this study, we recommend stereotactical normalization prior to SUSI analysis, although the effects of inter-subject variability of putamen size on diagnostic performance of the SUSI computed in patient space did not reach the level of statistical significance (possibly due to the rather small number of incorrectly classified cases).

Concerning the primary hypothesis put to test in this study (the classification performance of the SUSI relative to the SBR is better in the MULTI setting than in the MONO setting), the putaminal SUSI (computed in MNI space) performed slightly worse than the putaminal SBR in the MONO setting (AUC = 0.969 versus 0.976) and slightly better in the MULTI setting (AUC = 0.993 versus 0.991). This is in line with the primary hypothesis, although neither the difference in the MONO setting nor the difference in the MULTI setting reached statistical significance (*p* = 0.259 and 0.207, respectively). However, direct statistical comparison of the AUC difference between the SUSI and the SBR between the MULTI setting and the MONO setting showed a trend toward larger AUC difference in the MULTI setting compared to the MONO setting (*p* = 0.073, Fig. 6), supporting the primary hypothesis. Thus, we recommend the use of the SUSI in multi-site settings and in mono-site settings with more than one SPECT camera.

The fact that the SUSI did not outperform the SBR more clearly in the MULTI setting might be explained by two factors. First, very successful harmonization of SPECT image quality (including spatial resolution) in the PPMI by careful adaption of the acquisition protocol for each single camera and central image reconstruction at a core imaging center. Second, the relative IQR of the voxel intensity in the reference region was significantly larger in cases that were incorrectly classified by the putaminal SUSI compared to those that were correctly classified. The same was observed for the putaminal SBR. This demonstrates that the diagnostic performance of both, SUSI and SBR, is affected by uncertainty of the estimate of non-displaceable striatal FP-CIT count density by the count density in the reference region. The difference of the relative IQR between cases that were incorrectly classified by the putaminal SUSI and those that were correctly classified by the putaminal SUSI was larger in cases correctly classified by the putamen SBR compared to those incorrectly classified by the putamen SBR (Fig. 5). This is in line with the hypothesis that the impact of uncertainty in the estimate of non-displaceable count density is larger for the SUSI than for the SBR. However, the difference did not reach statistical significance. The small number of incorrectly classified cases might have contributed to the lack of significance.

Goethals and co-workers compared the SUSI computed in patient space and SBR computed in template space for detection of nigrostriatal degeneration in a mono-camera setting very similar to the mono-camera setting in the present study [16]. The AUC under the ROC curve for identification of neurodegenerative PS was highest for the SUSI of the whole striatum (minimum over left and right hemisphere, AUC = 0.859, 95% CI 0.766–0.952). The highest AUC achieved among several variants of the SBR was for the minimum over bilateral caudate and bilateral putamen SBR (AUC = 0.830, 95% CI 0.727–0.932). The minimum of putaminal SBR over both hemispheres was not considered in this study. The different ranking of SUSI and SBR in the study by Goethals and co-workers (striatal SUSI better than subregional SBR) compared to the present study (putaminal SBR better than striatal SUSI) most likely is due to methodological differences in the computation of the SBR.

The following limitations of the present study should be noted. First, the classification performance of SUSI and SBR in FP-CIT SPECT not only depends on the setting (multi-site/multi-camera versus mono-site/mono-camera). Classification performance of both semi-quantitative measures also depends on the subjects referred to FP-CIT SPECT.

The PPMI PD sample differs from the group of patients with neurodegenerative PS in the clinical mono-site/mono-camera sample in several respects, particularly by inclusion of patients with atypical neurodegenerative PS (MSA, PSP, CBD) in the clinical mono-site/mono-camera sample and the exclusion of subjects without evidence of dopaminergic deficit (SWEDD) from the PPMI PD sample [25]. There is also a relevant difference between the PPMI group of healthy control subjects and the non-neurodegenerative subgroup of the clinical mono-site/mono-camera sample: the latter included etiologies (e.g., vascular parkinsonism) that are associated with variable reduction of striatal DAT availability in a considerable fraction of patients. The differences between the PPMI multi-site/multi-camera research sample and the clinical mono-site/mono-camera sample most likely explain the fact that all tested semi-quantitative measures performed better in the PPMI sample than in the clinical sample despite the additional variability due to camera-specific effects in the PPMI sample.

Second, the excellent performance of both SUSI and SBR in the PPMI sample limits the power to detect performance differences (ceiling effect). Good diagnostic performance of FP-CIT SPECT independent of the analysis method in general makes it difficult to demonstrate differences in the diagnostic performance between methods and to reliably identify factors that might affect performance [2].

Third, classification performance of semi-quantitative measures also depends on the SPECT cameras, the acquisition protocol (including radius of rotation and delay of acquisition after tracer administration), the reconstruction algorithm, and, in the multi-site/multi-camera setting, the extent of harmonization of acquisition protocol and reconstruction algorithm. These factors not only affect the absolute classification performance of both SUSI and SBR, they also might affect the performance difference between SUSI and SBR. This may affect the generalizability of the results of the present study. In particular, it is to be expected that the SUSI more clearly outperforms the SBR in multi-site/multi-camera settings with lower degree of harmonization than in the PPMI.

## Conclusion

The specific uptake size index (SUSI) of striatal FP-CIT uptake is particularly appropriate for semi-quantitative analysis in multi-site/multi-camera settings in which camera-specific effects increase inter-subject variability of spatial resolution. SPECT images should be stereotactically normalized prior to SUSI analysis in order to reduce the impact of inter-subject variability of striatum size. The SUSI of the putamen provides better diagnostic performance than the SUSI of the whole striatum. In mono-camera settings without camera-specific inter-subject variability of spatial resolution, the specific binding ratio (SBR) provides similar performance as the SUSI. Errors of the estimate of non-displaceable count density can cause misclassification by both, SUSI and SBR, particularly in borderline cases. We recommend to check for high uncertainty associated with high variability of voxel intensity in the reference ROI in addition to verification of proper placement of the ROI.

## Abbreviations

AAL: Automatic anatomic labeling
AD: Alzheimer’s disease
APS: Atypical parkinsonian syndromes
AUC: Area under the ROC curve
BP: Binding potential
CBD: Corticobasal degeneration
CI: Confidence interval
DAT: Dopamine transporter
DLB: Dementia with Lewy bodies
FP-CIT: [I-123] N-ω-fluoropropyl-2β-carbomethoxy-3β-(4-iodophenyl)nortropane
FWHM: Full-width-at-half-maximum
HC: Healthy control
IQR: Interquartile range
MNI: Montreal Neurological Institute
MONO: Mono-site/mono-camera
MSA: Multiple systems atrophy
MULTI: Multi-site/multi-camera
NPH: Normal pressure hydrocephalus
OSEM: Ordered subset expectation maximization
PD: Parkinson’s disease
PDD: Parkinson’s disease dementia
PPMI: Parkinson’s Progression Marker Initiative
PS: Parkinsonian syndrome
PSP: Progressive supranuclear palsy
ROC: Receiver operating characteristic
ROI: Region of interest
SBR: Specific binding ratio
SPECT: Single-photon emission computed tomography
SPM: Statistical Parametric Mapping
SUSI: Specific uptake size index
VaP: Vascular parkinsonism
